# Legal issues in governing genetic biobanks: the Italian framework as a case study for the implications for citizen’s health through public-private initiatives

**DOI:** 10.1007/s12687-017-0328-2

**Published:** 2017-09-18

**Authors:** Cinzia Piciocchi, Rossana Ducato, Lucia Martinelli, Silvia Perra, Marta Tomasi, Carla Zuddas, Deborah Mascalzoni

**Affiliations:** 1 Faculty of Law, University of Trento, Trento, Italy; 2 Institut pour la recherche interdisciplinaire en sciences juridiques, Université Catholique de Louvain, Louvain-la-Neuve, Belgium; 3 MUSE – Science Museum, Trento, Italy; 4 Faculty of Economics, Law and Political Sciences, Department of Law, University of Cagliari, Cagliari, Italy; 5 Faculty of Economics, Free University of Bozen-Bolzano, Bolzano, Italy; 6 EuraC Resarch, Bolzano, Italy; 7 CRB, Uppsala University, Uppsala, Sweden

**Keywords:** Biobanking, Genetic data, Informed consent, Gene patent, Data protection, Privacy, Bankruptcy, General Data Protection Regulation, Italian law

## Abstract

This paper outlines some of the challenges faced by regulation of genetic biobanking, using case studies coming from the Italian legal system. The governance of genetic resources in the context of genetic biobanks in Italy is discussed, as an example of the stratification of different inputs and rules: EU law, national law, orders made by authorities and soft law, which need to be integrated with ethical principles, technological strategies and solutions. After providing an overview of the Italian legal regulation of genetic data processing, it considers the fate of genetic material and IP rights in the event of a biobank’s insolvency. To this end, it analyses two case studies: a controversial bankruptcy case which occurred in Sardinia, one of the first examples of private and public partnership biobanks. Another case study considered is the Chris project: an example of partnership between a research institute in Bolzano and the South Tyrolean Health System. Both cases seem to point in the same direction, suggesting expediency of promoting and improving public-private partnerships to manage biological tissues and biotrust to conciliate patent law and public interest.

## Introduction

The relationship between law, science and technology is a multifaceted interaction, which has increased in complexity over the last 20 years (Rodotà 1995; Jasanoff 1995; Brownsword 2008). Advances in bioinformatics and genomics and the possibility of access to several networks, infrastructures, and databases have reshaped our notions of doing biomedical research on the one hand (Trinidad et al. 2010) and on informational risk on the other, leading to the idea that anonymity in research may well be a chimera (Kaye 2012). The attempt to regulate research in genomics and biobank activities has impacted heavily on traditional legal concepts and categories such as property, privacy and informed consent (Kaye 2012; Kaye et al. 2015).

The studies of population genomics aim at understanding human health and gene environment interaction in the development of diseases, with the long-term goal of helping the discovery of targeted diagnostics and therapies. In order to achieve this objective, there is a need for large-scale collection of data on phenotypic traits (health data, lifestyle, behaviours) and wide availability of genomic data to carry out research into genetic variability and gene environment interaction across whole populations (Kaye et al. 2009; Mascalzoni et al. 2014a; Knoppers and Abdul-Rahman 2008). For these purposes, population biobanks have become a necessary infrastructure basis for life science innovation on an international basis and the recipient of considerable funding. The governance of biological materials and related data stored in genetic biobanks depends on the typology of the biobanks, on the anticipated use (different rules apply to the collections of biosamples used in different settings, as for instance in a clinical setting or for research, from patients or healthy individuals), on legal frameworks where nationally and internationally binding and non-binding rules apply and, last but not least, on the limits set by consent and agreements with individuals and groups.

As defined by the Council of Europe (2006), population biobanks are collections of human biological materials on a population basis derived from or destined for research projects, which contain “biological materials and associate personal data, which may include or be linked to genealogical, medical and lifestyle data and which may be regularly updated”. The large amount of samples and data required to achieve statistical significance has led, in the past decade, to a great increase in national and international biobanking creation, both commercial and public. Another phenomenon which has posed a great challenge to local and international regulation is the rise of worldwide consortia (GIANT, Rd-Connect, BioSHaRE, just to name a few) (Knoppers et al. 2011) aiming at information sharing (Budin-Ljosne et al. 2014) and at adopting common operating procedures, including approaches to ethical and legal requirements (Knoppers et al. 2014), such as consent (Gainotti et al. 2016), data protection (Budin-Ljosne et al. 2015) and privacy. Biological samples are collected from participants or patients, whose informed consent is needed for storage and use in genetic investigation, retaining an interest in the biomaterials and in the associated data. It is therefore controversial to apply traditional legal categories, such as “property” (Tallacchini 2005; Yassin et al. 2010) to determine the interests associated with biosamples, genetic data and the protection of research results linked to proprietary interests such as intellectual property or patents. Therefore, such traditional categories run the risk of being inadequate and new definitions, or at least new interpretations, are needed.

In setting up and formalizing networks of collection, storage and exchange of biomaterials and genetic data, biobanking activity has become a noteworthy example of the controversial relationship between research institutions and civil society, feeding into the wider debate on the oversight, governance, supervision and accountability of biological innovation. Indeed, the legal regulation of genetics biobanks is at the centre of different disciplines and opposing interests that need to be balanced.

One of the most controversial issues concerns the legal nature of the subject-matter of the biobanking activity, i.e. the biological sample (Macilotti 2013). The latter is a “res”, a tangible good, which traditionally attracts the category of property rights. At the same time, it is the carrier of genetic information that relates to the person who has donated that biological material. Furthermore, such information presents a challenging scenario, because its processing is likely to affect persons other than the data subject, namely his/her relatives and the whole biological family (Rodotà 2006). Groups and individuals, in fact, are linked by genetic information, even if they ignore their reciprocal family bonds, thus framing privacy in a new way (Mascalzoni et al. 2014b). It is therefore crucial that research activity is carried out taking into account the rights and the dignity of all subjects involved.

With regard to patients and research participants, genetics holds new implications for informed consent procedures. On the one hand, it allows for new possibilities but, on the other, it raises critical issues, such as the right to know or not to know, the right to obtain only partial information with appropriate counselling, the right to control the use of samples for a specific study, excluding other aims or investigations and the problems surrounding the return of incidental findings, family implications, international use and secondary uses of data (Burke et al. 2013; Budin-Ljosne et al. 2017).

Another critical point concerns the balancing between, on one side, property and intellectual property rights over biological collections and publications/inventions derived from them and, on the other, the public interest, i.e. the societal right to benefit from scientific progress.

Such competing demands illustrate the complexity of governing genetic biobanks and of drawing a line between public and private interests in this field. Here, public trust and civic engagement are particularly important issues, also bearing in mind the future-oriented features of this infrastructure which requires consensus on the governance of biological material deposited for future use and in the hands of brokers or an intermediary (often the biobank itself) supplying biospecimens to different researchers (Rothstein 2005) for different projects.

To manage controversial issues around the storage of biological materials of human origin, the Council of Europe has adopted recommendations (2016) oriented to “protect the dignity and identity of all human beings and guarantee everyone, without discrimination, respect for their integrity, the right to respect for private life and other rights and fundamental freedoms”. This updated version also contemplates provisions concerning the termination of a collection of biological materials: while it does foresee the possibility of giving consent for future uses, it suggests well-designed guidance to balance this freedom with the protection of individual rights, indicating the necessity for governance by the institution with the custodianship of the biomaterials, as well as clear rules for the access, sharing, and management of biosamples. Third party external review of projects carried out on biomaterials is required, as well as clear indications on possible return of results in general and on an individual level. Unexpected or planned closure, for instance depending from funding availability or bankrupt, in fact, raises important ethical, legal and social questions about the fate of stored biological materials and data, concerning the prospect of preserving, destroying or transferring them to other entities and, in this latter case, the transfer criteria, including the participants’ consent (Zawati et al. 2011). This issue has, over time, been a neglected aspect of biobanking regulation.

This paper will consider some of the gaps left by the interplay of different regulations that have led to paradigmatic examples in the Italian legal framework. In particular, we will consider the fate of genetic material in the event of insolvency of biobank and biobanks governance, as the outcome of a complex regulatory framework.

The first part provides an overview of the Italian legal regulation of genetic data processing, taking into account the entry into force of the new EU GDPR (General Data Protection Regulation), which seems to be research oriented and provides an opportunity to reach a fair balance between individual rights and research freedom, while the Italian regulation focuses on individual rights. The role of the data protection authority is taken into due account, providing a more flexible solution than the discipline left only to statutory law, which is the approach generally taken by civil law countries, such as Italy.

After this general outline, the article considers the fate of genetic material and IP rights in case of insolvency of biobanks’ and biobank governance as the outcome of a complex regulatory framework.

To this aim, we provide two case studies.

The first occurred in Sardinia and deals with the controversial bankruptcy case of one of the first examples of private and public partnership biobanks. This case offers the opportunity to analyse the special characteristics of biological samples, which are not to be regulated as “res” according to a traditional sense of the civil law. These characteristics have been addressed by the Italian data protection authority, which adopted some decisions blocking the processing of the biobank data. The paper considers different options to manage bankruptcy: in particular, it invites to consider the biological tissues as commons to be owned by a public biobank or, as an alternative, to be managed according to new kind of partnerships which allow a balance between commercial purposes, the respect of participants, and the public interest.

Finally, we provide for a second case study, the Chris project, as an example of partnership between a research institute in Bolzano and the South Tyrolean Health System, which aims at guaranteeing the interaction between researchers and the local population and to ensure that in any event there is a public funding responsibility to sustain the project which involves the local population.

We suggest that, while some changes in the regulations could account for resolving some of these cases, others could be covered only by a well-structured collaboration between law, governance and self-regulation. In fact, while the legal landscape alone sometimes does not provide satisfactory answers, alternative governance models that use mixed models (including self-regulation) can offer possible flexible solutions.

### The Italian regulatory framework on genetic data processing and the impact of the General Data Protection Regulation: balancing interests?

The processing of genetic data in Italy is governed by a regulation that is, in many respects, stricter than those existing in other EU Countries. This situation will most likely change, with the entry into force of the new EU GDPR. This act, approved in May 2016, will repeal Directive 95/46/EC with effect from 25 May 2018: this two-year period will allow Member States to revise or adapt their legislation in order to comply with the GDPR. This shift in paradigm from a Directive (setting certain aims to be achieved) to a Regulation (setting specific rules to be complied with) can be referred to as a ‘harmonization through adaptation’ process (de Hert and Papakonstantinou 2016). The Regulation offers renewed focus on the protection of “sensitive data”, including both health and genetic data, and on research taking advantage of information from registries, which “can provide solid, high-quality knowledge which can provide the basis for the formulation and implementation of knowledge-based policy, improve the quality of life for a number of people and improve the efficiency of social services” (Recital 157).

For the sake of clarity, the level of harmonization pursued by means of the Regulation, is actually an “incomplete” one. Article 9.4. of the GDPR, in fact, allows Member States to “maintain or introduce further conditions, including limitations, with regard to the processing of genetic data, biometric data or data concerning health”. This allows Member States to introduce further appropriate and possibly differentiated safeguards for the rights and freedoms of the data subject.

Traces of the attention paid by the GDPR to scientific research and biobanking activities, although not fully clear, can be read in different provisions.

First, from the general viewpoint of the scope of the Regulation, with regard to the functioning of biobanks, Art. 4(3b) introduces a definition of “pseudonymisation”, which is defined as “the processing of personal data in such a manner that the personal data can no longer be attributed to a specific data subject without the use of additional information, provided that such additional information is kept separately and is subject to technical and organisational measures to ensure that the personal data are not attributed to an identified or identifiable natural person” (Article 4(5)). Recital 26 states that Personal data which have undergone pseudonymisation, being attributed to a natural person by the use of additional information, should be considered to be information on an identifiable natural person and fall within the scope of the Regulation. Nonetheless, the scope of identifiability is qualified by the reference to “means reasonably likely to be used”: therefore, there may be cases where pseudonymised data, together with a combination of organisational, legal and technological measures, can be considered anonymous data (Knoppers and Saginur 2005; Wjst 2010; Bolognini and Bistolfi 2017). This is still a matter to be clarified and, along with other questions related to data for research, is under discussion by different groups comprised of researchers, policy makers and patients’ group representatives which, as provided for under the GDPR, will propose a code of Conduct for research to the commission, an effort lead by the BBMRI ERIC (http://www.bbmri-eric.eu/bbmri-eric/).

Secondly, the new Regulation recognizes that it is often impossible to fully identify the purpose of personal data processing for scientific research purposes at the time of data collection. Therefore, in keeping with recognized ethical standards, data subjects should be allowed to give their consent to certain areas of scientific research. In a departure from the 1995 Directive, where consent provisions were often narrowly interpreted, Recital 33 creates an opportunity to bring consent closer to a broad consent model, currently employed in the research practice by some Member States. The possibility of consenting to “certain areas of scientific research” allows detachment from an informed consent clearly tailored to a specific research project.

Another aspect, which once again stresses the favour shown to research, is related to secondary uses. Generally speaking, the Regulation prevents personal data collected for one purpose being used for another incompatible purpose. However, some provisions explain that further processing for scientific research, statistical or historical purposes can be considered “not incompatible” purposes. In order to benefit from this presumption specific safeguards must be fulfilled (Safeguards are set out in Article 89 and Recital 156, as well as in Article 9, when data concerning health is processed).

The awareness and trust towards research and biobanking shown by the GDPR marks the most evident general difference between the Italian discipline on these issues and the EU approach. It is important to highlight that the current version of the GDPR is the result of negotiations and the first draft was very strict regarding secondary uses.

In Italy, the regulation on the processing of genetic data has been left to the Italian Data Protection Authority (IDPA) which places great emphasis on individual rights. The Italian legislator, when reorganising the whole discipline of privacy, decided to refer to an independent administrative authority for the identification of rules for regulating the processing of genetic data. According to Art. 90 of the Italian Data Protection Code (Legislative Decree no. 196 of 30 June 2003, IDPC) the processing of genetic data, regardless of the entity processing them, is allowed exclusively in the cases provided for in ad-hoc authorisations granted by the IDPA in agreement with the Minister for Health and the *Istituto Superiore di Sanità* [Higher Health Care Council]. Genetic data is thus considered as “hyper” sensitive information, subject to a special set of rules (the possibility of using and disseminating personal data for research purposes is regulated by another Authorization (no. 9/2016) which explicitly excludes genetic data from its scope), provided by the General Authorization for the Processing of Genetic Data (no. 8/2016). This document provides for general principles (also involving the management of biological samples—solely considered as a source of information) concerning the purposes of use, requirements (i.e. consent), storage and communication.

Although scientific research is explicitly recognised as a legitimate purpose for the processing of genetic data (upon consent given by the data subject), the lack of specific provisions for certain types of research activities is easily identifiable.

A couple of examples will clarify the point. With regard to informed consent, the General Authorization states that “genetic data may be processed and biological samples used exclusively for the purposes specified herein, on condition the person concerned has provided his/her written informed consent thereto” and “information notices shall include (…) a detailed list of all the specific purposes to be achieved” (Points 5 and 6). This provision clearly disregards the possibility of developing research tracks following further purposes, not fully identifiable at the time of data collection. Furthermore, the processing of genetic data for purposes other than those for which the personal data were initially collected is only possible where the scientific and statistical purposes are *related directly* to those for which the data subjects’ informed consent had been obtained initially. Samples and data can be used only for different research projects (without re-consent), in cases where the data subjects can no longer be identified or if, despite all reasonable efforts, it is impossible to contact the data subject and the research program has been specifically authorized by the IDPA and given a favourable opinion by an ethics committee (Point 8). As always, one solution does not fit all. Re-contact is a very sensitive issue, as it is costly and burdensome, and needs to be pursued only when necessary, but when necessary should be pursued (Black et al. 2013; Green et al. 2013; Budin-Ljosne et al. 2017; Budin-Ljosne et al. 2011; Gainotti et al. 2016; McCormack et al. 2016).

This quite strict framework, not supported by any provision on how re-consent can be obtained easily, is further completed by the provision on “Data Communication and Dissemination” (Paragraph 9), which can be divided into two parts. Under the first, “Genetic data may not be communicated and biological samples may not be made available to third parties unless this is indispensable for the purposes mentioned herein” or under specific contracts that ensure the same degree of security under the data controller. The method of “communicating” data may concern the possibility of working on joint projects together with other research institutions, possible only through complex Data Transfer Agreements. Moreover, unidentifiable data can be communicated for the pursue of *directly related* aims, but no communication or transfer to third parties is generally allowed. The second part of this paragraph states: “[n]o genetic data may be disseminated. Research findings may only be disseminated as aggregated information, or in accordance with such arrangements that can prevent data subjects from being identified also by way of indirect identification data; this shall also apply to publications”. Even consent seems incapable of overcoming this broad and general prohibition, considering that—where deemed feasible—other points of the Authorization explicitly refer to individual consent as a way of lifting restrictive prohibitions. Besides seriously hampering the possibility of sharing individual research data (in particular on public accessible genetic databases), this provision, taken together with others, demonstrates the precautionary attitude of the whole Italian regulatory framework regarding the processing of genetic data.

Time will tell how the Italian legal system will confront the opportunity offered by the GDPR, and if it will in some way take advantage of the “partial” harmonization strategy allowed by the EU, introducing higher standards of protection for the individual and thus modifying the balance struck by the Regulation.

Understanding this background is a necessary precursor for correctly identifying and contextualizing the case studies that will be described in this paper.

The problem with a strict law where complexities are incompletely grasped is that in some cases it may end up hampering scientific research without fulfilling its purpose: protecting individuals, as highlighted in the first case.

### Case study n. 1: the Sardinia case

Sardinia is the second biggest island of Italy, located right in the middle of the Tyrrhenian Sea. In some of its areas, geographical isolation and historical factors resulted in development of genetically homogenous populations. Talana village in the central-eastern Ogliastra area, for instance, was established a thousand years ago by a remarkably small number of original settlers. Among the 1200 inhabitants who were the subjects of a census at the beginning of 2000, 75% descended from 8 founder fathers and 10 founder mothers, and 95% of marriages involved spouses of the same village, 35% even consanguineous. Moreover, church records enable families to be traced back to the 17th century (Meldolesi 2000). This population can rightly be regarded as a genetic isolate, characterized by a homogeneous genetic background and therefore suitable for analysing the genome-environment and lifestyle interaction. Because of antiquity, slow demographic growth, isolation and high degree of endogamy and consanguinity, i.e. the most amenable features for genetic population studies, Sardinia (with particular focus on its isolated areas) has since the middle of 1990s been an ideal research location for accomplishing genetic population studies aimed at identifying multifactorial genetic traits with potential biomedical interest. Among these features longevity, since Sardinia has been designated a “Blue Zone”, i.e. a location with the highest numbers of centenarians in the world (Poulain et al. 2004). Substantial projects and ventures were developed. Worth mentioning is one of the first research studies carried out in the Ogliastra area, the multidisciplinary AKEntAnnos project by the Institute of Population Genetics of the Italian National Research Council (IGP-CNR), aimed at identifying genes predisposing to multifactorial diseases and to complex traits, under the epidemiological, genealogical, genetic, molecular and statistical profiles (Deiana et al. 1999). To unravel the genetic processes involved in age-related traits and diseases, the Italian National Research Council (CNR) Institute of Cagliari and the US National Institute on Ageing (NIA) carried out the SardiNIA project, also called ‘Progenia’ for the Sardinian public (https://sardinia.nia.nih.gov/).

In 2000, the “genetic exceptionalism” of the Ogliastra region also facilitated the creation of one of the first Italian public and private partnerships in genomics: Shardna. Founded as an Italian S.r.l. (limited company), Shardna soon attracted various investors, both from the private and the public sector. The Shardna project aimed at establishing a primary resource for identifying genes for complex diseases, such as hypertension, kidney stones, migraines, obesity, eye diseases and hair loss (Meldolesi 2000), involving 10 villages of the mountainous region of Ogliastra (Baunei, Escalaplano, Loceri, Perdasdefogu, Seui, Seulo, Ussassai, Urzulei, Talana and Triei). A critical mass of biological samples from 11,700 individuals was collected, giving rise to a genetic biobank, publications (complete list available at http://web.tiscali.it/shardna) and patents. Inspired by a forward-looking vision, research was carried out during the whole project in close collaboration with the local communities, generating a virtuous circle of participation based on informative meetings, a careful design of truly informed consent and constant contact with field staff and researchers. A laboratory and a small clinic were set up in the selected villages, and as a benefit, participants could receive individual feedback on the genetic analysis and free medical check-ups (Artizzu 2008). This latter was an appealing motivation since the nearest hospital was located an hour away from most of the villages in the Ogliastra district. Thanks to this active engagement of the population and the constant information provided in loco by the researchers, the percentage of volunteers was very high, surpassing 80% (Artizzu 2008).

Nearly 10 years after its creation, a transition occurred at the top of Shardna company, giving rise to a controversial bankruptcy case, which is worth analysing as a case study for its weight of social, legal and ethical implications. In 2009, the majority shareholder sold his shares for 3,000,000 Euros to the Fondazione San Raffaele, a prestigious Italian foundation involved in scientific and medical research and health and charitable activities. In 2011, however, the San Raffaele Foundation was hit by a financial crisis and forced to start a procedure of arrangement with creditors (in Italian “concordato preventivo”), opening the door to the bargain sale of Shardna by a bankruptcy court in 2012 (see the decree of the Tribunal of Milan at: http://www.sanraffaele.org/static/upl/de/decretoomologa10-05-2012.pdf). After years of silence about the outcome of the procedure, finally in July 2016 the media (see for instance: ANSA 2016; The Guardian 2016) spread the news on the purchase of Shardna by Tiziana Life Sciences Plc, a UK biotechnology company (http://www.tizianalifesciences.com/Welcome_to_Tiziana.html). Tiziana Life Sciences created Longevia genomics Srl, an Italian subsidiary, in order to assign the Shardna assets. Despite an estimate value of 3,000,000.00 Euros, Tiziana Life has finally bought it for just 258,000.00 Euros. The bankrupt assets include the right to use the biological samples plus the clinical documentation; the declarations of consent of the participants; the equipment and the content of the biobank; the database comprising the medical histories of the donors. The declared goals of the newborn company are to continue the Shardna project as well as to start its own new research activities (Italian Data Protection Authority, *Provvedimento di blocco del trattamento dei dati personali contenuti in una biobanca*, 06.10.2016).

The events surrounding Shardna’s bankruptcy have resonated beyond Italy, being the subject of a Parliamentary question to the Commission in July–August 2016 (see Question for written answer to the Commission by Giulia Moi (EFDD), P-005318-16 and the answer given by Ms. Jourová on behalf of the Commission, 25 August 2016, P-005318/2016). They have also been followed with great attention by the Italian Data Protection Authority (IDPA). As recognized under Italian law, the relevant case law (for instance, the case S. and Marper v UK decided by the European Court of Human Rights), and the new General Data Protection Regulation, biological samples share a common ground with personal data. Indeed, biological samples are the tangible manifestation of an informational content: they are the carrier of personal information, which is ontologically embedded in them. In other words, biological samples are able to reveal information which refers to an identified or identifiable person. For this reason, they deserve the same level of protection reserved to personal data.

As a consequence, the biobank transfer should be guided by the relevant applicable provisions concerning data protection: personal data must be processed fairly and lawfully, collected for specified, explicit and legitimate purposes, not be further processed in a way incompatible with those purposes and not kept longer than necessary for those purposes. In particular, Article 16 of the IDPC (Legislative Decree 196/2003) contains a specific provision concerning the issue at stake. It establishes that, in the event of processing termination, for whatever reason, the data shall be alternatively: (a) destroyed; (b) assigned to another data controller, provided they are intended for processing under terms that are compatible with the purposes for which the data have been collected; (c) kept for exclusively personal purposes, without being intended for systematic communication or dissemination; or (d) kept or assigned to another controller for historical, scientific or statistical purposes, in compliance with laws, regulations, Community legislation and the codes of conduct and professional practice. However, this provision has to be coordinated with the above-mentioned General Authorization for the Processing of Genetic Data (no. 8/2016) which allows the transfer of biological samples to third parties (not involved in joint projects) only where certain conditions are met (see the previous paragraph).

With reference to the Shardna events, over the last few years the IDPA has received hundreds of donors’ complaints, alleging in particular the lack of information regarding the conservation period of genetic data and biological samples; the role of Parco Gen.O.S. in the processing and the identity of the data controller of the biobank (see IDPA, *Provvedimento di blocco del trattamento dei dati personali contenuti in una biobanca*, 06.10.2016). Furthermore, complaints also concerned with unlawful collection of personal data from the municipal data archive, the lack of appropriate security measures, and the inability of donors to exercise their rights (for instance, the withdrawal of consent and the right to consent to the processing performed by the new data controller).

On the basis of a preliminary assessment, the Authority has considered that Tiziana Life Sciences aims to undertake processing compatible with the purposes for which personal data were originally collected. However, according to the IDPA, “the facts present some critical issues with regard to certain aspects of the processing” (Italian Data Protection Authority, *Provvedimento di blocco del trattamento dei dati personali contenuti in una biobanca*, 06.10.2016). For this reason, pending completion of the Authority’s investigation, with Decision 389 taken at the beginning of October 2016, our national data protection Authority has established the immediate blocking of the processing of the biobank data. This means that Tiziana Life Sciences must refrain from any further processing of data and biological samples, apart from ensuring the appropriate storage of the latter; re-contacting data subjects to provide information and acquire new consent; providing adequate responses to data subjects who want to exercise their rights. Therefore, it is expected that the coming months will be crucial for the fate of the Sardinian biobank.

### … and its legal consequences: what happens in case of insolvency of biobank? A private-public perspective

In the Italian legal order, the problems underlying the treatment of genetic material, and especially the protection of genetic information increase in the context of the crisis of a company and of its subsequent bankruptcy. The reason for this difficulty lies in the absence of a unitary legislation precisely regulating the limitations of the use of genetic material and identifying its owner, once the genetic material is separated from the source subject. Last but not least, a further problem lies in the classification of a biobank and, especially in the case of bankruptcy, in the relation existing between the classification and the type of company or the legal system.

The bankruptcy of the Sardinian company, previously mentioned, gave us the opportunity to think about the legal fate of genetic material in case of the insolvency of biobanks and, in particular, the possibility of forming the subject-matter of a bankruptcy sale. According to the Italian regulation which is, as stated above, quite incomplete on this point and, according to the majority of legal scholars, in the event that the genetic material could not be transferred, in particular because of the lack of specific authorization given by participants to the biobank. Despite this, the official receiver has not only handled the data base sale (including all the genetic data collected over years), but has also handled the numerous blood test-tubes protected in cold storage (refrigerating rooms).

This decision caused public controversy regarding the official receiver’s powers in the bankruptcy centre and determined two different actions. They have been respectively undertaken, the first one before a Court, for the suspension of the bankruptcy sale, and it has not yet been determined, and the other on the administrative side before the IDPA for the suspension of the processing of personal data.

What are the decisional powers held by Administrator following the failure of the company holding the biobank and, particularly, as regards liquidation of the company assets?

The Italian bankruptcy law, in sections of Art. 104 and s. 1. which regulates the procedure of liquidation of social assets after bankruptcy, states that the official receiver has to use every competitive method in the sales operations so that he/she can sell the assets of the company to the highest bidder (Vassalli et al. 2014). This means that according to the Italian bankruptcy law there are no requirements placed on buyers in relation to the property sold; likewise, it is not possible to know how this property will be used once sold. In other words, the official receiver cannot favour any buyer: this means that the material could be sold to entities who pursue a (even partially) different purpose, not only from that of the biobank, but also from the purpose which the donor had authorized in the consent form (Zawati et al. 2011; Carroll 2002, Janger 2005).

In this specific case, indeed, the official receiver, aware of the necessity of finding a specialised buyer through focused search, decided that the winning bidder should, as the main object of his/her activity, have conducted research activity in the genetic-medical field.

Therefore, how has the need for the donor’s authorisation been set alongside the gap in the Italian legal order in case of bankruptcy of a company which holds genetic material? First of all, the collected and catalogued genetic material should be considered as a vehicle for both personal information and potential public interest in pursuing the research aim. In this case, the whole set of EU rules concerning the protection of sensitive data and the management of biological samples may be applied. Specifically, the official receiver should be bounded by Article 16 IDPC and, in particular, by the obligation to assign the data to another data controller only if they are intended for processing under terms that are compatible with the purposes for which the data have been collected or for historical, scientific or statistical purposes. In this way, the official receiver has to choose potential buyers who will be able to adhere to the scientific aim of the research. This use should be in compliance with the indication contained in the consents given by participants, unless new consent can be obtained. In case a correspondence is not found and re-consent is not obtained, the “extrema ratio” would be the destruction of the collected samples. However, this solution would not be workable for at least two reasons: first, because this would harm the bankruptcy assets; secondly, because it would destroy a valuable resource which could be further exploited for research purposes. To avoid this event, appropriate consent forms are required, where the patient can expressly decide about the transfer of the biological material to the biobank to pursue a specific scientific aim, even in case of company failure or bankruptcy. Nevertheless, there is no evidence that, in the Sardinian case described, any authorization was given. If this were the case, this situation might complicate the bankruptcy sale and the pursuit of the research: indeed, the winning bidder could not have any legitimacy in maintaining the research activity.

A different option in managing biological materials in the event of liquidation/failure of a biobank, could be found by considering human tissues as commons, therefore enhancing the value of the information contained in the biological sample, and transforming the sample itself into an asset, which can be identified by a court. If we accept that perspective, it would be necessary to identify an entity or a recognised organisation able to guarantee both the nature of the biological samples as commons and the respect of the aim of the research through their proper use. This entity could be a public institution, specifically an entirely publicly owned biobank which keeps the tissues and the information, sharing the samples with the researchers who ask for them. In this perspective, the publicly owned biobank would function not only as a guarantor of privacy and as a protector of the scientific research, but it would also be free from any risk regarding bankruptcy events, not being the type of business entity subject to such risk.

The acceptance, in civil law legal systems as well, of the model of a charitable trust (biotrust) would be an interesting development, in order to regulate the functioning of biobanks, with clear governance available for scrutiny of participants at the time of the collection, where individuals could assess whether they may trust the model for access, sharing and managing contact, re-contact etc. (Winickoff and Winickoff 2003). The structure of this model would, on the one hand, be able to monitor the observance of the duties of the researchers and, on the other, to promote the engagement of the participants in the biobank in the management of scientific research. Additionally, one-time consent seems obsolete, in an era of fast development and the nature of research, which changes day by day.

All in all, many of the problems dealt in this paragraph could be easily solved through the precautionary choice of a form of association which is not subject to insolvency procedures and by ensuring transparent information at the time of decision making.

### The patentability of human genes in the framework of European and Italian regulation

The patentability of human genes is another interesting topic to provide an analysis of the social implications of public-private initiatives in the field of genetics.

Both a direct and indirect commercial value is associated with biological samples, information about sample donors and inventions resulting from research with samples and associated data. Directly, through the patenting, under certain conditions, of DNA sequences; indirectly, where the study of aggregate samples results in the development of new medicines and personalized care techniques.

The economic value of genetic resources corresponds to how they are assessed as potential sources of profit, represented by the utility, applicability and reproducibility within the market of chemical and biological information which such resources contain. Information deriving from biological diversity, once elaborated in the research and development phase, acts, in turn, as a basis for new products and, therefore, can be considered a double resource: a resource “per se”, and a new primer for innovative research. Nevertheless, conflicting interests between freedom of scientific research and access to scientific knowledge are now arising, requiring a more careful equilibrium between the public and the private domain (Reichman et al., 2016).

Currently, in Europe, the legal protection of biotechnological inventions involving genetic material is ensured by the framework of Directive 98/44/EC (Directive 98/44/EC of the European Parliament and of the Council of 6 July 1998 on the legal protection of biotechnological inventions (GUCE, L 213 of 30 July 1998)) and the European Patent Convention (EPC). On the other hand, the international discipline of patents is represented by the Agreement on Trade-Related Aspects of Intellectual Property Rights (TRIPs 1994). The TRIPs Agreement sets minimum standards that member States must comply with when regulating IPRs in their domestic systems. In the majority of the European Union member states, the provisions of Directive 98/44/EC have been included within different national laws regulating patents. As regards Italy, the directive’s content has been included in the Industrial Property Code within Legislative decree no. 131 of 2010.

The Italian legislation (Industrial Property Code, Legislative decree, n. 30 of 10 February 2005) provides the patentability of an invention relating to an isolated element of the human body or otherwise produced by means of a technical process, even if its structure is identical to that of a natural element (Art. 81-quater lett. d) industrial property code, in accordance with Arts. 3, 4, 5.2 dir. 98/44/EC); in particular, when the biotechnological patent concerns genes or sequences of genes, there is an obligation to indicate and specifically describe its function and industrial pertinence in the patent description and in the claims of the patent application.

In addition, according to Article 170-bis, paragraph 3, of the Italian Industrial Property Code, “a patent application relating to an invention whose object is, or that utilizes, biological material of human origin, must be accompanied by the express, free and informed consent, for that sample and utilization, of the person from whom the material was taken, based on applicable legislation” (see Article 170-bis of the Italian Industrial Property Code, and Article 22.5 of the Implementing Regulation of the Italian Industrial Property Code) (Rovati 2016).

The aforementioned provision must be read in conjunction with three further provisions: article 22, paragraph 5, of the Implementing Regulation of the Italian Industrial Property Code, and articles 170-bis, paragraph 7, and 173, paragraph 7, of the Italian Industrial Property Code. The following conclusion can be drawn from a thorough reading of such provisions: (i) the patent applicant must attach the statement of consent to the patent application; (ii) the consent must concern both the sample-taking and the subsequent use of the biological sample; (iii) where the statement of consent has not been attached, the Italian Patent and Trademark Office (UIBM) sets a date for integrations and further observations, and only if the office feels it cannot accept these observations it rejects the application. Finally article 170-bis does not seem to address whether the statement of consent should cover only the commercial use or even use for experimental purposes.

However, the patent system does not deal directly with the issue of consent acquisition to the sample-taking and of the use of data and biological material; this profile is in fact regulated by different legislation and based on various international and constitutional rules (see Art. 3.2 Charter of fundamental rights of the European Union; arts. 2, 13, 32 Italian Constitution; Arts. 16 and 22, Oviedo Convention on Human Rights and Biomedicine; Arts. 6 and 8 International Declaration on Human Genetic Data 2003. See also Romandini 2007).

Furthemore, we have to take into account that a biobank is constituted of an organized collection of biological material (such as samples of blood, blood serum, plasma, DNA) and corresponding database containing data and information related to the samples (and the donors). In this sense, it has been widely debated whether the biobank (as collection of biological samples) may be considered as a database (Bygrave 2012) and, in this latter case, whether it is eligible for protection under copyright laws or the sui generis right. Under European and Italian Law, a collection of works, data and other materials can be defined database when it is: (i) independent; (ii) systematically or methodically arranged; (iii) individually accessible. If the above-mentioned requirements are met, and provided that the database is creative, then copyright protection is granted; on the contrary, if the requisite of creativity lacks, but the creation of the database has required a substantial economic investment, it is entitled to the sui generis rights protection. The evaluation of the level of originality of the biobank database must be done in a case-by-case basis. However, considering the object of the activity of a biobank, it will be probably hard to configure its database as an original one. Indeed, for the purpose of collection and study, the selection and arrangement of the contents is generally a trivial one: samples and data are arranged according to pathology, put in a chronological/alphabetic order, etc. On the contrary, if there is a substantial investment in either the obtaining, verification or presentation of the contents, the maker of the database can enjoy the sui generis right protection. However, the application of this kind of protection to the specific context of biobank is still controversial (Ducato 2013).

What is the fate of the intellectual property rights owned by the biobank if it goes bankrupt? As regards such rights, these are freely transferable by contract, either definitively or temporarily. In fact, and in the event of bankruptcy, these economic rights become part of the entire pool of the company’s assets (Maffei-Alberti 2009; Spolidoro 2002), and therefore, in the organization of a biobank, any software developed for the management of data and information will be freely transferable. Such software is also eligible for copyright protection under International, European and domestic law. On the other hand, as regards the biobank’s personal data contained in the database, it will be subject to the stricter rule of Article 16, paragraph 1, lett. b), of the Italian Data protection law (Legislative decree no. 196/2003).

According to this provision, in the event of data processing termination, “the data shall be […] assigned to another data controller, provided it is intended for processing under terms that are compatible with the purposes for which the data has been collected”.

One last clarification concerns the ownership of intellectual property rights for Italian law. In this respect, it must be recalled that such rights arising from a patent for invention or copyright, even where the invention or work has been created at the expense of a company, do not belong to the legal entity, which acquires ownership in a derivative way from the physical person, who has actually realized the invention or work. This is a substantial difference with respect to copyright and patent legislation of the common law IPRs system, where a company may be originally the owner of intellectual property rights.

As regards the topic subject of analysis, we underline the existence of two different centres of interests. On the one hand, private companies demand the right to enjoy full protection of their patented inventions, inclusive of the several forms of economic exploitation. On the other hand, whether or not the human genome is a public good is heavily debated, hence not available for private exploitation. This tension should be reconciled, considering several benefits that both public and private actors may enjoy when they look for synergies. For instance, States may pursue a policy of commercialising scientific research, whereas private companies should pay attention to the positive externalities deriving from a cooperative attitude, such as corporate reputational gains and increased employee morale. Such an approach, represented by the creation of public-private partnerships, has proven to be effective in another sensitive field where patent law and public interest clash, namely access to essential medicines in developing countries (Pusceddu 2014).

### Case study n. 2: the CHRIS study: towards a partnership research/community

“The Cooperative Health Research In South Tyrol (CHRIS) study is a population-based study with a longitudinal lookout established in 2011 to investigate the genetic basis of common chronic conditions associated with human ageing, and their interaction with life-style and environmental factors in the general population of South Tyrol” (Pattaro et al. 2015).

All individuals 18 years + and older from middle and upper Vinschgau/Val Venosta are invited to participate to the study. Ten thousand participated in the study so far. Family participation is encouraged for complete pedigree reconstruction and disease inheritance mapping. The goal of the CHRIS study is to investigate the interaction between the genetic basis of common chronic conditions with lifestyle and environmental factors in the general population. The CHRIS study is focused on cardiovascular, metabolic, neurological and psychiatric health. Seventy-three blood and urine parameters are collected for deep phenotyping and 60 aliquots per participant are collected and preserved in the CHRIS Biobank Samples are genotyped on one million variants with the Illumina® technology.

The CHRIS study has a longitudinal design plan, with follow-up starting after 6 years from initial recruitment. In addition, the CHRIS study is being conducted in the same geographical area where the MICROS study was previously carried out in 2002/03 (Pattaro et al. 2015).

The need for follow-ups and re-contact is embedded in the design of big population studies such as HUNT in Norway, the Framingham study near Boston etc. The possibility to re-phenotype or follow-up according to preliminary results to enrich the dataset and follow new research lines is a desirable setup, often blocked by demanding and pricy logistics. In CHRIS, the need to re-contact and establish a stable setup was clear from the beginning, leading to a strategy aimed at building long-lasting trust within the community and the stakeholders. This aim is also reflected in the recruitment strategy and the ELSI approach to the study as well as in the management of the data and the bioresources.

The communication with the community and the individuals happens in different times along the development of the study and is especially rich during the recruitment phase. Embedded in the community on many levels, the CHRIS study is a public private partnership between non-profit Academia and the South Tyrolean Health care system. The recruitment centre is located in the valleys’ reference hospital of Schlanders/Silandro, the central town of the valley. In each municipality, the recruitment begins after an informative communication campaign comprising sharing the study concept with local general practitioners meeting local authorities and the leaders of local charities and voluntary organizations; announcing the study to the population through the local media and holding a town hall public meeting where the study is officially introduced to the community. This last step guarantees direct interaction and discussion with the public, allowing the time and opportunity for questions. The active part of the recruitments foresees a direct invitation by a personal letter mailed to all 18 + -year-old inhabitants (addresses identified through publicly available electoral lists). To favour the identification of genetic variants that might be enriched in single families, entire families are encouraged to participate. For this reason, the first invitation is mailed personally to each member of the same family at the same time followed by up to two reminders.

The study is organized to enroll up to 10 participants/day.

After the informed consent procedure, participants undergo tremor assessment, blood drawing, urine collection, anthropometric measurements, electrocardiographic (ECG) analysis and blood pressure measurement. Finally, participants respond to a computer assisted personal interview and a computer aided self-interview.

One week later, participants receive a letter with the complete results of their clinical assessments, including blood, urine and 10-s ECG results validated by a clinician. Participants are invited to discuss the results with their GP. Laboratory life-threatening findings are followed up through an emergency protocol which, via the study coordinator and the reference GP, guarantees that the participant is alerted in the shortest possible time. A senior medical doctor and the emergency department of Schlanders/Silandro hospital are covering necessary immediate interventions due to serious cardiac issues occurring during the ECG or problems arising during blood drawing according to the study’s emergency protocols.

The Ethical and legal framework in CHRIs tries to follow a participant-centric approach.

The CHRIS study was approved by the Ethical Committee of the Healthcare System of the Autonomous Province of Bolzano (19 Apr 2011). In addition, the CHRIS study invested in creating a comprehensive ethical, legal, and social implication (ELSI) framework aimed at building and ensuring long-lasting trust and participation.

The study is compliant with current Italian and EU regulation and with the Helsinki Declaration. Privacy and security in data handling and sharing are strictly enforced and a public access code regulates how data and samples can be used. Data and samples are only shared for specific projects and based on Material and/or Data Transfer Agreement (Pattaro et al. 2015).

The CHRIS governance comprises different levels: an internal committee which monitors everyday issues (data and sample access, study management) and three oversight external bodies: the ethical board, the scientific board and an evaluation committee that evaluates the project’s major changes and includes stakeholders from the local healthcare system and study participants. Legal and ethical issues are described in the Ethical and Legal regulation published on the study website and include aspects such as return of results, duties and management of the study, custodianship and benefit-sharing policies in case of revenues coming from the research, what happens if the study terminates etc. The whole governance and the Ethical and Legal regulation are publicly available and provide a transparent policy for the community and the stakeholders.

To this end, CHRIS implemented a dynamic consent model that comprises dynamic information strategy and an IT tool to support changes over time.

Given that the CHRIS study is designed to be longitudinal, with use of data and samples that will be extensive and prolonged over time, an interactive dynamic consent process for empowering participants’ autonomy and complying with current regulations was also implemented. Dynamic consent includes two important parts: an ongoing information section and an interactive consent webpage with dynamic options where individuals could choose between different setups.

Information is provided in different formats to improve understanding through the use of diverse media that replace the information sheet. After booking the appointment, participants receive at home (by post or email) a detailed information brochure (http://www.chrisstudy.it), which includes a description of the study, images illustrating key concepts in lay language, and all ethical and legal issues relevant for the informed consent process. At the study centre, the participant is invited to watch a 9-min information video (available on the study webpage) that systematically and fully explains the project. The video shows the whole research workflow, outlines how data and samples are handled, what security measures are in place and what the risks involved are, and it describes the participant’s rights and information sources through images and small animations. After viewing, the participant can ask questions of the study assistants. While it was not meant to replace oral communication between participant and study assistant, the introduction of the video had the effect of shortening the time needed for further explanations from about 20 min before its introduction to less than 5 min after its implementation. A yearly newsletter and updated information on the webpage complement the ongoing information for consent.

After the video, electronic consent is filled in online directly on the personal interactive consent webpage. The type of consent asked for is broad with regard to the aim of the study. At the same time, the consent is layered and provides dynamic options (changeable online over time) regarding data sharing (international, public data repositories), return of secondary/unexpected results (outlining the right to know or the right not to know) and the permission to use samples and data in case of death or if the subject loses legal capability.

The data regarding access levels granted by each participant goes directly into the database and ensures that data can automatically be filtered for different purposes according to the participants’ choices.

The dynamic tool can also be used for re-contact, collecting additional information and re-consent, should this be necessary in the future.

Direct measurements and blood parameters are returned to participants but no financial compensation or travel cost reimbursement is offered to support participation.

Return of unexpected secondary or health threatening results is provided upon prior explicit participant’s agreement to be re-contacted. In this case, a multistage consent will take place so that the participant can be properly re-contacted. In fact, in the event of genetic clinically relevant findings, an agreement with the Health Care System genetic counseling unit ensures that participants are approached by a medical geneticist, who undertakes proper counseling before results are tested and confirmed.

Follow-up information flows through the annual newsletter providing information about new developments and through the personal webpage in case of important developments that require direct interaction, such as re-consent.

### Access to the bioresource

Sample management, operation, and monitoring instruments are integrated in a Biobank Information Management System (BIMS) to ensure security of the biobank collection.

Access to the bioresource is regulated through an access committee which evaluates research protocols asking to access data and samples and based on an access regulation for internal and external use. The biobank has joined the Biobanking and Biomolecular Resources Research Infrastructure (BBMRI) which provides protocols that guarantee top-level biological and medical research by promoting procedure standardization and sample quality. In order to maximize transparency on the use of samples and data and for tracking the use of the bioresource, the CHRIS biobank was assigned a “Bioresource Research Impact Factor” (BRIF) code (http://www.p3g.org/brif-bioshare-pilot-study): BRIF6107.

### EURAC South Tyrol healthcare partnership

Biomedical research is needed to identify factors that affect the aging process, which may lead to preventive interventions for healthy aging with reduction of health care related costs. For this reason, the CHRIS study was established as a collaboration between a research institute (the EURAC Center for Biomedicine) and the South Tyrolean Health System. Such a collaboration guarantees that the study operates by actively interacting with the local population, thus raising awareness towards a more conscious approach to health. The study is expected to foster a dynamic cycle among scientists, clinicians and the whole population, which is to offer reciprocal feedback to ultimately improve individuals’ health.

The partnership ensures that the biobank and the Data Resources, in case EURAC were not able to sustain it further, are under “public” funding responsibility, ensuring a degree of sustainability for the bioresource that so much invested in the community and for the community.

Currently, the genetic epidemiology community is facing the issue of data sharing on a big scale. Population-based research is pressured by the dichotomic need on the one hand to protect data privacy and security, on the other hand, to maximize the use of stored data and samples, so as to guarantee the maximal benefit to the community. Data harmonization, pooling, and sharing are beneficial to scientific research provided that data security and privacy are guaranteed as new tools and governance suggestions have been developed to foster both aspects, beyond the law (BioSHaRE and DATASHIELD).

By promoting clear governance rules, third parties’ assessment with regard to data and samples access and, at the same time, by maintaining open an individual choice level (dynamic consent tool), the challenge to adapt to new scientific aspects and simultaneously comply with upcoming regulations seems more feasible.

## Conclusion

In this paper, we have considered a series of case studies, in the light of the legal framework of genetics biobanks in Italy.

We have focused on the European and Italian legal framework, analysing in particular the entry into force of the new EU General Data Protection Regulation (Regulation (EU) 2016/679 of the European Parliament and of the Council of 27 April 2016) and its impact on the Italian legal system. In addition, we have provided an analysis of the regulation of gene patenting, balancing the economic value of genetic resources with freedom of scientific research and access to scientific knowledge.

We took into consideration two case studies, dealing respectively with the Sardinia genetics database and the CHRIS project, which provided specific insights into the governance of biobanks’ in Italy. The first one, Shardna, concerned the legal fate of genetic material and data in case of the biobank’s insolvency, where a static governance did not take into account some possibilities of flexible adaptability, and we concluded that there is a need to strike a balance between private interests surrounding collected genetic material and public interest to their proper use and scientific research. In this regard, we concluded that an entirely publicly owned biobank might guarantee all the interests at stake. As an alternative, the model of the biotrust will be a particularly interesting and challenging solution to explore in a civil law country such as Italy.

Still, the development of science with the compelling need for re-contact and feedback results is increasingly demanding an adaptable governance model that does not respond only by setting regulations. The second case shows how a self-created governance, supported by tools to enable participatory input and with the support of dynamic IT-based consent provided the participants with the ongoing freedom to decide and change their mind about the fate of data and biosamples, thus accounting for re-contact needs by researchers and, at the same time, for the changing nature of research.

These two case studies demonstrated that a civil law country such as Italy had to provide for flexible legal tools, to manage all the ethic, legal and societal implications of biobank regulation and governance. Beside, although the topics and the case studies we took into consideration are different from each other, they all seem to point in one direction, urging the need for cooperation and synergies between public and private actors, law and soft provisions. Public-private partnership might respond to the converging interests—public and private, economic and scientific research oriented—and to the need for flexibility of regulation of these complex objects, which are the biological samples and the precious information they contain.
